# Diagnostic Communication and the Care That Follows: A Critical Narrative Review

**DOI:** 10.3390/diagnostics16193211

**Published:** 2026-10-03

**Authors:** Theodoros Xanthos

**Affiliations:** School of Health and Care Sciences, University of West Attica, 12243 Athens, Greece; theodorosxanthos@yahoo.com

**Keywords:** diagnostic communication, diagnostic labelling, nocebo effects, incidental findings, informed consent, diagnostic revision, patient safety, shared decision-making

## Abstract

An accurate diagnosis does not ensure that a patient understands its implications or receives appropriate care. This critical narrative review draws on purposively selected literature studies on diagnostic labels, explanations of test results, nocebo effects and care after diagnostic revision. Terminology experiments show changes in perceived severity and stated preferences, often in hypothetical settings. Clinical effects on symptoms and healthcare use depend on the intervention, comparator and outcome; explanation is frequently combined with treatment changes. Nocebo experiments demonstrate possible symptom mechanisms, not the cause of an individual patient’s continuing symptoms. Studies of diagnostic follow-up identify a separate problem: accurate or revised findings may fail to inform subsequent care. The synthesis distinguishes evidence for changing understanding, relieving symptoms and completing appropriate clinical action. A documented misunderstanding or obsolete instruction can warrant correction while symptom assessment continues. Evaluation should measure accurate understanding, informed choices and care received, with symptoms assessed separately. Truthful disclosure and follow-up remain responsibilities even when symptom benefit is uncertain.

## 1. Introduction

A radiology report may correctly describe disc degeneration while leaving the patient expecting progressive disability. A small thyroid lesion may seem more urgent when it is called cancer. A negative investigation may be taken to exclude every cause of a symptom when it has excluded only one. In each case, the clinician needs to explain what the result means for this patient: what it establishes, what may happen next and what care is appropriate. Studies of diagnostic labels and imaging reports show that diagnostic wording can change perceived severity, interpretation and preferences even when the finding itself is unchanged [1,2,3].

A better explanation should help the patient understand the result and decide what to do next. Understanding may improve while pain persists. Immediate relief from anxiety may be less helpful if the patient mistakenly believes that further assessment is unnecessary. Researchers therefore need to describe what was said or written, what other care changed and what the explanation intended to achieve. Changes in anxiety, treatment preference, pain and service use each tell us something different.

Nocebo research is relevant because a diagnostic explanation may influence symptoms through expectations and learning. Under controlled conditions, negative expectations and learning can increase pain and other symptoms even when the physical stimulus is unchanged [4,5]. Other consequences of a diagnosis arise through the care it permits or prevents: it may authorise treatment, alter a referral, validate a person’s experience or restrict access to a service. A patient advised to avoid activity may follow that advice regardless of whether expectations increase the pain. Both the effects on symptoms and the consequences for everyday activity and care need attention.

For many patients, receiving a diagnosis is helpful. Naming an illness can explain longstanding symptoms and provide access to support. The same label may frighten someone whose finding is mild or incidental; qualitative research records both relief and concern [1,6]. When a diagnosis is removed, clinicians also need to consider what the patient may lose, including an explanation they have relied on or a route to care, even when the correction is justified.

Communication is already part of established accounts of diagnostic quality. The National Academies’ definition of diagnostic error includes failure to communicate the diagnostic explanation to the patient [7]. In diabetes care, Ledford and colleagues’ qualitative model of shared meaningful diagnosis connects the diagnostic label with information transfer, patient interpretation and later management [8]. The proposed Rumpelstiltskin effect likewise brings naming, meaning, expectations and learning together to explain possible therapeutic consequences of receiving a diagnosis [9]. These precedents and the reviews of labels and imaging communication [1,2,3] make it necessary to ask what the evidence adds beyond the general proposition that explanations matter.

This review compares the evidence needed to justify three different actions: clarifying understanding, treating symptoms and ensuring that revised findings inform care. Evidence that a label changes stated preferences differs from evidence that shows symptoms improve; correcting a record differs from a change in subsequent treatment. A documented misunderstanding or unsupported restriction can warrant correction while symptoms remain under investigation. The clinical applications are the author’s synthesis and require evaluation.

## 2. Review Approach

This critical narrative review examines how diagnostic communication affects understanding and decisions, symptoms, and subsequent care. Diagnostic communication includes explanations to patients, reports used by clinicians and the transfer of revised findings between services. Thematic searches of PubMed-indexed records through Europe PMC began in August 2026 and were repeated during manuscript development, through 23 September 2026. Targeted searches and reference-list checking supplemented this process. Recent English-language studies were considered alongside earlier foundational work. Here, critical denotes examination of which inferences a study design and its measured outcomes support, rather than a numerical quality rating or an assumption that all communication is beneficial.

A supplementary theme-by-theme literature search was conducted on 30 September 2026 using web searches of publicly indexed bibliographic records, publisher pages and institutional repositories. Search concepts covered diagnostic labels and terminology, imaging and reassurance, incidental findings and notification, nocebo education, diagnostic uncertainty, de-labelling, variant reclassification and results-guided management. Supplementary File S1 records the executed search strings, source-access limitations and examples of the evidence considered. This update supplements the initial search; it is not a reconstruction of its historical queries or a systematic search of additional bibliographic databases (Supplementary Materials).

Literature selection remained purposive and interpretive rather than exhaustive. Trials, experimental studies, qualitative and observational research, reviews and relevant guidance were considered. For the synthesis, priority was given to sources directly addressing a diagnostic explanation, label or revised finding and a defined patient or care outcome. Controlled clinical comparisons informed clinical-effect claims; hypothetical experiments and healthy-volunteer studies informed narrower claims about responses or mechanisms. Qualitative studies informed experience and implementation questions, while reviews and guidance supplied context and professional standards. Selection did not depend on statistical significance: null, mixed and potentially adverse findings were considered alongside benefits. The clinical domains were retained to compare naming and choices, interpretation of reports, expectation-related symptoms, and the transfer of revised diagnoses into records and care, not to represent all specialties. These contrasts also distinguish a change in knowledge from a change in delivered care.

Additional targeted searches examined diagnostic uncertainty, communication needs, access to care and potentially adverse responses, including mistrust. Supplementary File S1, under Section S7, records these searches and the evidentiary roles of the sources considered. Findings were examined for absent benefits, divergent outcomes and unintended effects, without restricting eligibility to studies labelled ‘negative’. Studies without a diagnostic communication intervention could inform context about access or utilisation, but not estimates of communication effects. These targeted checks do not constitute a new systematic database search.

**Figure 1:**
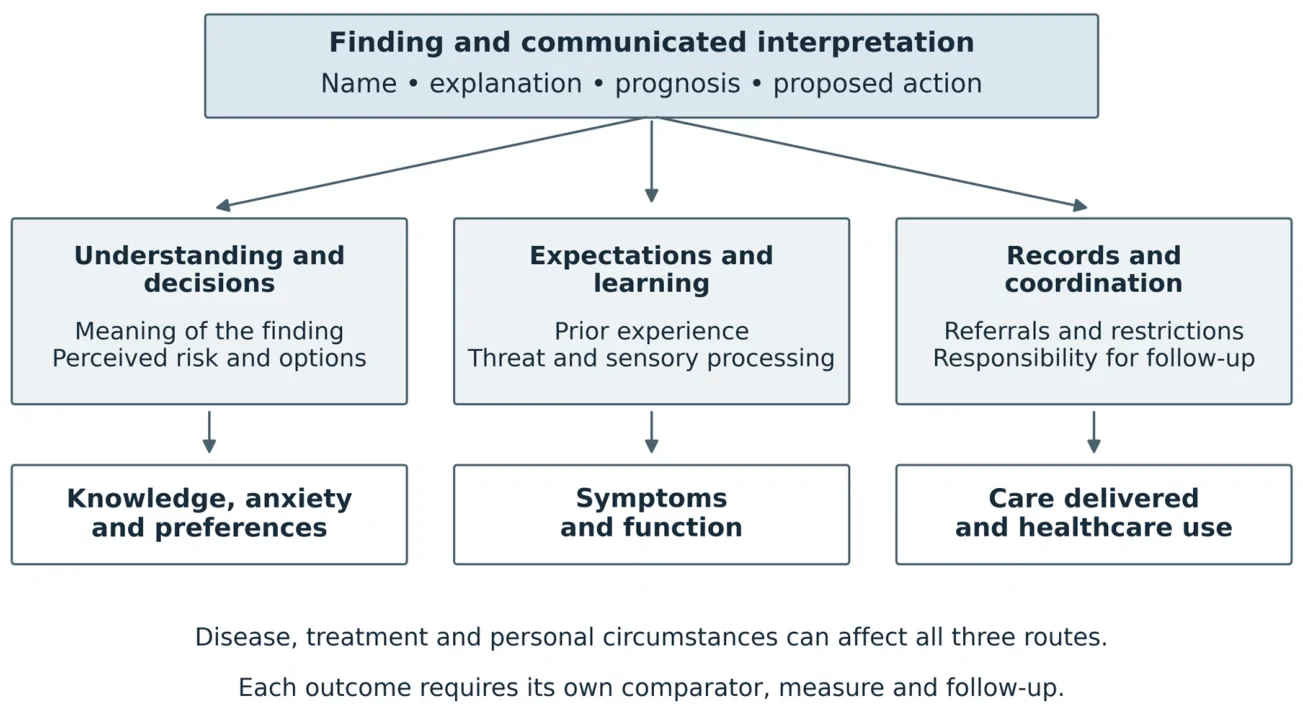
How diagnostic communication may affect patients and care: an author-derived analytic framework. Understanding and decisions, expectation-related changes in symptoms, and coordination of care may influence one another. The columns identify questions to assess, rather than independent or exclusive pathways. Arrows are conceptual and do not represent measured effects or a validated causal model. Disease, treatment and personal circumstances may influence all three areas.

The three outcome domains represent an interpretive organisation of the selected literature, not a prospectively specified framework governing study identification or eligibility. The distinction was already used in the initial narrative synthesis; its definitions and outcome-based assignment rules are made explicit here. Findings are assigned according to what was measured: understanding and decisions; symptoms and function; or subsequent care. A study can inform more than one domain. Perceived severity, self-rated understanding and assessed comprehension are distinguished, and neither stated preferences nor record updates are treated as completed clinical actions. The framework is analytic, not a validated causal model; its clinical applications are separate proposals derived from the comparison. Figure 1 illustrates this distinction, while Table 1 identifies the evidence supporting each clinical premise and separates it from suggested practice.

Critical appraisal considers the population and recruitment setting; whether participants faced their own diagnosis or a hypothetical scenario; allocation and blinding where relevant; the recipient of the information; the comparator; accompanying treatment or organisational changes; outcome definition and measurement validity; primary versus secondary analyses; follow-up; attrition; and clinical applicability. Effect estimates and confidence intervals are considered alongside their clinical importance and precision. A non-significant comparison is not treated as equivalence. Intervention packages are interpreted as packages unless their components can be separated. Observational associations are not assigned the causal force of randomised comparisons, and qualitative findings are not used to estimate effect sizes. Weighting is therefore question-specific and qualitative, not a pooled or numerical hierarchy. No meta-analysis, formal risk-of-bias tool or numerical quality score was used. The resulting clinical suggestions are not evidence that the proposed combination of actions is effective.

## 3. Effects of Diagnostic Communication

### 3.1. Diagnostic Labels and Treatment Choices

Studies of terminology ask how people respond when the same condition is given different names. Nickel and colleagues’ 2017 review identified seven such studies, all using hypothetical scenarios [2]. Descriptions that sounded more medicalised or serious generally increased perceived severity, anxiety or preference for intervention. Random allocation supports a causal effect on these immediate responses. Whether people would make the same choices about their own care remains uncertain: a clinical decision also involves discussing alternatives and facing the consequences of treatment.

Perceived severity is a judgement of seriousness; self-rated understanding expresses confidence in understanding; assessed comprehension requires evidence that a person can correctly explain prognosis, options or uncertainty. These are distinct outcomes. A change in perceived severity or anxiety does not establish improved comprehension, and accurate understanding can coexist with concern. The terminology experiments should therefore be read as evidence about the responses actually measured, not as demonstrations of better-informed decisions.

One thyroid study tested both repeated choices and the response to the first description a participant encountered. The crossover experiment included 550 Australian adults without a history of thyroid cancer. Total thyroidectomy was chosen by 108 (19.6%) when the condition was called papillary thyroid cancer, 58 (10.5%) when it was called papillary lesion and 60 (10.9%) when it was called abnormal cells. The first-exposure analysis instead counted the choice of either form of surgery, with 60/186 (32.3%), 46/191 (24.1%) and 47/173 (27.2%), respectively. Both risk-ratio confidence intervals comparing the alternative names with cancer included one. At first exposure, diagnosis-related anxiety was 0.8 points lower with papillary lesion than with cancer on a scale from one to 11 (95% CI, 0.3–1.3 points lower). Responses also depended on the order in which descriptions were presented [10].

Because the crossover involved repeated hypothetical choices, its larger differences should not be used to predict how much a naming policy would reduce surgery. The first-exposure analysis also counted a broader surgical outcome. In a separate discrete-choice experiment with 2054 adults, terminology influenced the harms participants were willing to accept to avoid a thyroid cancer death [11]. Language clearly belongs in discussions of treatment choice, but these experiments do not establish which name helps patients make better decisions about their own diagnosis.

Two online factorial experiments analysed responses from 1340 men and 1435 female partners after randomising 1402 and 1493, respectively; participants who did not finish the survey were excluded [12]. The label and benefit–harm information were varied separately. Label effects depended on the information provided (interaction *p* = 0.02 in men and *p* = 0.009 in women). With limited information, ‘lesion’ rather than ‘cancer’ reduced women’s preference for curative-intent treatment by 11.6 percentage points (95% CI, 4.3–18.9 points lower). More detailed information within the cancer-label group reduced that preference by 10.3 points (95% CI, 2.9–17.7 points lower); the corresponding estimate in men was uncertain. Neoplasm-versus-cancer comparisons at a fixed information level were also uncertain.

Compared with cancer plus limited information, neoplasm plus detailed information reduced curative-intent preferences by 9.2 points in men (95% CI, 3.3–15.1 points lower) and 12.6 points in women (95% CI, 5.2–19.9 points lower). The results therefore concern combinations of name and explanation. These were hypothetical choices in separately recruited samples, with post-randomisation exclusions, not observed treatment decisions or a randomised comparison between sexes.

The value of a diagnosis also depends on the patient’s circumstances. In an online experiment involving 181 young women, adding polycystic ovary syndrome (PCOS) to an otherwise comparable scenario increased perceived severity and intention to undergo ultrasound, and reduced self-esteem [13]. Interviews with 26 women who reported a clinical PCOS diagnosis described a wider range of experiences. Some welcomed an explanation for troubling symptoms. Others with milder symptoms recalled shock and concern about future illnesses. Fertility worries sometimes influenced contraception or pregnancy planning [6]. These interviews identify questions about fertility and prognosis that deserve discussion, although they cannot establish that the label caused each subsequent decision.

Anxiety after a diagnosis may also change with time. A systematic review of 16 studies in asymptomatic people receiving non-cancer screening diagnoses found increased anxiety soon after labelling, with limited and inconsistent evidence of longer-term effects. Findings at three months depended on the meta-analytic model, while depression, general mental health and absenteeism did not deteriorate uniformly [14]. Early distress may call for explanation and support; the evidence does not justify describing it as permanent psychological harm.

Diagnostic explanations have also been studied in functional neurological disorder. A 2026 systematic review included 15 studies and found that structured or reinforced communication was often associated with better understanding and satisfaction. Evidence for acceptance, recovery and healthcare use was inconsistent [15]. The studies address how to explain an established clinical diagnosis. They provide no basis for equating functional neurological disorder with nocebo or attributing the disorder to the conversation in which it was diagnosed.

A change in name sometimes follows a change in how a disease is understood. In the original study of non-invasive follicular thyroid neoplasm with papillary-like nuclear features (NIFTP), all 109 patients with non-invasive tumours were alive without evidence of disease at a median of 13 years after surgery [16]. These postoperative findings contributed to removing the carcinoma designation from a strictly defined pathological entity; the diagnostic criteria remain essential [17]. They do not describe what would have happened to untreated lesions. Proposals to change terminology for low-risk ductal carcinoma in situ (DCIS) similarly require evidence about natural history and diagnostic boundaries, as well as evidence about patients’ responses to the name [18].

### 3.2. Imaging Findings and Reassurance

A spinal abnormality on imaging needs to be interpreted in relation to the patient’s symptoms. Degenerative findings are common in people without symptoms [19]. They may matter in a patient with pain, but their presence alone does not establish the pain mechanism, predict deterioration or justify avoiding ordinary movement. Patients want to know what the result means for prognosis, treatment and self-management [20]. Replacing a technical word with a familiar one may leave those questions unanswered.

In the randomised low back pain scenario study, less structural-sounding labels reduced perceived need for imaging, but most activity and work beliefs changed little [21]. Because accompanying explanations also differed, this was not a test of names alone. An earlier online experiment found that altered report wording, with or without epidemiological information, improved an unvalidated composite of back-related perceptions; the enhanced reports did not differ significantly [22]. Neither study measured recovery or actual activity, and an additional benefit from epidemiological information was not established. Table 1 summarises the diagnostic-labelling comparison.

In a small clinical MRI trial, 44 patients received either a factual explanation of their report or reassurance that the findings represented normal changes. Pain, self-efficacy and quality-of-life outcomes favoured reassurance at six weeks [23]. The subsequent systematic review rated the evidence as low certainty [3]. This was a comparison of explanatory approaches, not an isolated word substitution.

The Lumbar Imaging with Reporting of Epidemiology (LIRE) trial added prevalence information to clinician-facing reports. It found no significant difference in the primary utilisation outcome and a small difference in a secondary opioid-prescribing outcome that was attenuated when other prescribers were included [24]. The secondary endpoint replaced a planned dose measure because dose data were unavailable (Table 1). Patient understanding and symptoms were not measured; the prescribing result therefore does not establish a patient-mediated nocebo mechanism.

PREVENT estimated the additional effect of intensive education on active listening, both alongside first-line care, rather than the value of all explanations or reassurance [25]. A small 2026 trial of brief positive-versus-negative messages detected no immediate differences in physical performance or pain [26]. Its power calculation targeted a large strength effect, so smaller effects and equivalence in pain remain unresolved (Table 1). Neither trial tested repeated explanations of a patient’s own imaging findings.

Reassurance is also inconsistently measured. A scoping review found that only one of 46 trials asked directly how reassured patients felt, using an unvalidated item [27]. Reduced fear or fewer consultations cannot simply be equated with reassurance. Rolfe and Burton’s review found no clear overall improvement in worry, anxiety or persistent symptoms after diagnostic testing in selected low-risk populations [28]; its numerical findings are in Table 1. These results do not remove the need to investigate plausible serious disease or explain what a negative result leaves uncertain.

**Table 1:** Selected studies, classified by evidence setting. The recipient, comparator and measured outcome determine the inference; estimates across rows are not directly comparable.

Evidence Setting; Study and Population	Comparison and Follow-Up	Principal Finding	Interpretation
Hypothetical: Thyroid terminology; 550 adults without prior thyroid cancer [10]	Randomised crossover scenarios; immediate choices and anxiety.	At first exposure, surgery was chosen by 32.3% (cancer), 24.1% (lesion) and 27.2% (abnormal cells). Risk ratios versus cancer: 0.73 (0.53–1.02) and 0.82 (0.60–1.14).	Crossover responses favoured less-invasive options with alternative names; first-exposure surgery comparisons were inconclusive. Actual treatment was not measured.
Hypothetical: Low-back-pain labels; 1375 participants [21]	Randomised scenarios; diagnostic names and explanatory content varied; immediate beliefs and intentions.	Perceived need for imaging: approximately 4.2–4.4 versus 5.7–6.0/10. Most activity/work beliefs differed little, but degeneration increased agreement that the person should not work.	Effects differed across beliefs. The trial did not measure imaging use, return to work or recovery.
Clinical: LIRE; 238,886 analysable adults undergoing spine imaging [24]	Stepped-wedge cluster trial; prevalence information added to clinician-facing reports; one year.	Spine-related relative value units: −0.7% (−2.9% to 1.5%). Opioid prescription from a study clinician: adjusted 36.2% versus 37.0%; odds ratio: 0.95 (0.91–1.00), *p* = 0.04.	No significant primary utilisation difference. The broader-prescriber analysis was inconclusive. The secondary prescription endpoint replaced a planned dose measure; symptoms and patient understanding were not measured.
Clinical: PREVENT; 202 patients with acute low back pain and elevated risk of persistence [25]	Intensive education versus active listening, both with first-line care; primary pain outcome at three months.	Pain difference: −0.3/10 (−1.0 to 0.3).	No clear additional pain benefit over active listening. This comparison does not estimate the value of all explanations or reassurance.
Clinical experiment: Verbal messages; 46 adults with chronic primary low back pain [26]	Standardised negative versus positive messages before physical and functional tests; immediate assessment.	No between-group differences detected in strength, functional performance, pain intensity or pressure pain thresholds.	Brief exposure; sample-size calculation targeted a 0.75 standardised strength effect. Smaller effects and equivalence in pain are not resolved.
Hypothetical: Brain MRI report; 803 online participants [29]	Original versus AI-simplified report; immediate comprehension and anxiety.	Assessed comprehension: 5.61 versus 5.78 on a score ranging from 0 to 7 (*p* = 0.007). Anxiety did not differ significantly (*p* = 0.103).	One hypothetical report in an online sample. A small comprehension gain was not accompanied by lower anxiety.
Clinical synthesis: Diagnostic testing; 14 trials, 3828 patients at low risk of serious disease [28]	Testing strategies assessed for reassurance, symptoms and subsequent consultations.	Persistent symptoms beyond three months: odds ratio at 0.99 (0.85–1.15; ten trials). No clear overall benefit for illness worries or anxiety.	Testing showed no clear overall benefit for illness worries or anxiety in these selected low-risk populations. These findings do not address the value of testing when serious disease remains plausible.
Clinical: CorMicA and ILIAS ANOCA; 151 and 153 patients, respectively [30,31]	All participants underwent coronary function testing. Results disclosed with tailored management versus results withheld with standard care.	SAQ summary score benefit: 13.6 points (7.3–19.9) at one year and 9.4 points (3.9–14.9) at six months, respectively.	The trials tested use of the findings to guide care. They do not estimate testing alone or separate explanation from changes in management.
Clinical: CorCTCA; 231 randomised patients [32]	Results disclosed versus blinded. Primary outcome: diagnostic reclassification; symptoms and satisfaction were secondary outcomes.	Adjusted SAQ difference: −3.76 points (−8.79 to 1.27) at six months; −2.06 (−7.27 to 3.14) at twelve months. Diagnostic classification and global treatment satisfaction improved.	No demonstrated angina benefit; symptom follow-up was incomplete. Neither equivalence nor a between-trial difference is established.
Implementation: Follow-up notification; 2548 patients with actionable incidental findings [33]	Additional telephone, letter or portal notification versus an existing tracking system alone; within one month of the follow-up due date.	Completion: telephone, 60.4%; letter, 58.7%; portal, 36.4%; control, 53.2%. Telephone alone significantly exceeded control (*p* = 0.014).	Channel preference and completed follow-up differed. The findings concern a specific tracking system and do not establish the best notification channel for other workflows.
Implementation: ALABAMA; 823 adults with recorded penicillin allergy [34]	Allergy assessment, behavioural support and record updating versus usual care; up to twelve months.	First-line penicillin prescribing: 72/401 versus 14/410; adjusted relative risk: 5.27 (3.03–9.18), absolute difference: 14.21 percentage points: (9.92–18.49).	Primary outcome amended after pandemic disruption. Prescribing improved; treatment response failure did not differ significantly. Neither a communication-only effect nor unrestricted de-labelling is established.
Implementation: PROPEL; 2052 adults across twelve inpatient units [35]	Stepped-wedge trial of staff education and electronic-record tools; primary endpoint at discharge.	Label removal: 45/1018 versus 31/1034; adjusted odds ratio: 2.05 (1.08–3.91).	Label removal improved, but adjusted penicillin-use comparisons were not significant during admission or at three and eighteen months.

Values in parentheses are 95% confidence intervals unless otherwise stated. Lower pain scores and higher Seattle Angina Questionnaire (SAQ) scores indicate improvement. CorCTCA differences are intervention minus control. Abbreviations include: AI, artificial intelligence; ANOCA, angina with non-obstructive coronary arteries; MRI, magnetic resonance imaging.

### 3.3. Incidental Findings and Access to Results

Patients with an incidental finding need to know why observation, further testing or no further action has been recommended. In the baseline survey accompanying Watch the Spot, 2049 people responded, approximately 5.9% of the enrolled trial population. At six to eight weeks, 32.2%, 9.4% and 7.2% of respondents reported mild, moderate and severe nodule-related distress, respectively. People who had not been notified promptly by a clinician reported greater distress and anxiety [36]. The low response rate limits what these proportions tell us about the whole cohort. The association nevertheless identifies a practical concern for services: how long patients wait between detection of a finding and an explanation of what it means.

A quality improvement project gave a fact sheet to 101 patients with 3–8 mm solid nodules. Afterwards, 84/101 reported less anxiety, and self-rated understanding increased from 4.2 to 8.1 on a scale from one to 10. The project used unvalidated measures and had no concurrent control [37]. This is perceived understanding, not demonstrated comprehension of prognosis or surveillance. Studies of letters and interviews during nodule surveillance describe the questions patients want answered: what is a nodule, why is surveillance advised, and how will the next assessment be arranged [38,39]? Answering these questions remains part of care even when some concern is appropriate.

Readable reports may improve comprehension without relieving anxiety. A review of 18 studies found frequent improvements in perceived understanding or satisfaction, but limited evidence on decisions or behaviour; none measured recall, and twelve used hypothetical reports [40]. In the online brain MRI experiment, a small gain in assessed comprehension was not accompanied by a significant reduction in anxiety [29] (Table 1). These are different outcomes, neither of which establishes better clinical decisions.

The possibility of worry does not mean that patients prefer to wait for results. In a four-centre survey, 8139 of 43,380 invited portal users responded (18.8%). Of those who answered the preference question, 7520/7859 (95.7%) wanted immediate access; the proportion was 2337/2453 (95.3%) among respondents with non-normal results [41]. These preferences describe respondents, not all patients. A scoping review nevertheless records both the value of access and the difficulty of interpreting results without timely professional help [42]. Services need to consider how to offer that help alongside access, rather than assume that potential distress means a patient would choose delay.

Responsibility for results extends beyond sending a message. Wright and colleagues’ rapid review, practice interviews and patient consultation identified unclear responsibility and fragmented information transfer as recurring obstacles to follow-up [43]. In a separate quality improvement study, detailed record review classified 518 of 593 analysable radiology recommendations (87.4%) as resolved. This included both completed actions and documented clinical decisions that an action was unnecessary. Expert reviewers judged 31 unresolved cases to pose substantial risk [44]. In a retrospective pulmonary nodule study, recommendations entered through a dedicated tool specified the imaging modality and interval more completely than recommendations sent through the usual alert system [45]. These studies concern care processes, not cancer mortality. Their immediate implication is to assign responsibility for reviewing a finding and documenting the follow-up decision.

In the randomised notification trial, adding telephone contact to an existing tracking system improved follow-up completion; letters did not significantly improve completion, and the portal group had the lowest completion despite preference for that channel [33] (Table 1). Preference, receipt of information and completed care therefore require separate assessment. The result concerns a particular workflow and does not establish that portals are generally inferior.

These findings distinguish access to information from its clinical use. Patients may understand a report better and still be worried; appropriate surveillance may require more visits rather than fewer. Whether the explanation also changes symptoms is a further question, to which nocebo research contributes a different kind of evidence.

### 3.4. Nocebo Mechanisms and Their Clinical Limits

A nocebo effect occurs when negative expectations or learning in a healthcare context cause an adverse change in symptoms or experience. Symptoms reported after a warning, or during placebo treatment, may also reflect illness, natural variation or other influences. Experiments test whether the context contributes by changing what participants are told or have learned while controlling the physical stimulus or treatment [4,46].

A systematic review of 37 experiments in healthy participants found effects on pain and itch. For pain, pooled Hedges’ g was 0.71 (95% CI, 0.60–0.82) for conditioning combined with verbal suggestion and 0.63 (95% CI, 0.40–0.86) for suggestion alone. These estimates came from different sets of studies and did not provide a randomised comparison of the two methods [5]. In observational-learning experiments, watching another person’s response also changed pain ratings and expectations in healthy observers [47]. When clinicians ask about a patient’s expectations, earlier treatment and other people’s experiences may therefore be relevant as well as the current explanation.

Experimental nocebo effects have also been accompanied by changes in the nervous system. In a spinal functional MRI study, an inert cream used in a nocebo procedure increased pain ratings and pain-related activity in the ipsilateral dorsal horn relative to a control condition with the same heat stimulus [48]. Neurobiological reviews describe threat-related and pain-modulatory networks, including prefrontal, cingulate and insular regions and descending influences on nociception [49]. This evidence supports a biological basis for contextual effects on pain under experimental conditions. It does not provide a clinical biomarker showing that a particular diagnostic conversation harmed a patient.

Experiments also examine persistence and reversal. Kunkel and colleagues detected conditioned nocebo effects at one-week follow-up in healthy participants [50]. In Meijer and colleagues’ study, open-label counterconditioning reduced responses more than extinction; an advantage for closed-label counterconditioning appeared only in the sensitivity analysis restricted to nocebo responders [51]. Verbal suggestions also differed, and equivalence between open- and closed-label procedures was not established. These experiments motivate clinical investigation but do not establish the effectiveness or expected speed of recovery after correcting a diagnostic explanation.

Visible anxiety alone gives little basis for attributing a symptom to nocebo. In a meta-analysis of 59 independent studies, experimentally induced negative expectancy and state anxiety were associated with larger nocebo effects; dispositional anxiety was not significantly associated with the effect [52]. A frightened patient may have misunderstood a prognosis, accurately understood a serious threat, experienced repeated dismissal or developed a new illness. Asking what the patient fears helps distinguish these possibilities without assuming that fear explains the symptom.

### 3.5. Assessing a Possible Nocebo Contribution

If a clinician suspects a nocebo contribution, the assessment should identify the symptom, the warning or learned association thought to be relevant, and their timing. A warning cannot explain the onset of a symptom that preceded it, although it may have influenced later severity or persistence. Other possibilities include worsening disease, treatment effects, altered activity and increased attention to an existing symptom. Persistent physical symptoms may involve several interacting processes, including established disease [53]. A restriction recorded in a referral may help explain reduced activity or blocked access to care. That is a reason to review the restriction, regardless of whether expectations have affected pain.

Repeated comparisons within the same person can sometimes help separate treatment effects. SAMSON randomised 60 people who had stopped statins because of symptoms from periods of statin, placebo and no tablets; in total, 49 completed the full year. Mean symptom scores, on a scale with a maximum of 100, were 16.3 during statin periods, 15.4 during placebo periods and 8.0 during no-tablet periods. The statin–placebo difference was not statistically significant [54]. Comparing these periods helps distinguish exposure to the drug from the experience of taking tablets. However, participants knew when they were taking no tablets, and the study did not isolate expectation as the sole explanation. In StatinWISE, 151 of 200 randomised participants contributed to the primary analysis. The mean statin–placebo difference in muscle symptoms was −0.11 on a scale from zero to 10 (95% CI, −0.36 to 0.14) [55].

These trials gained information from repeated comparisons within individuals, but the pooled results do not show that every participant’s symptoms were unrelated to the drug or that every symptom was a nocebo effect. Diagnostic information is harder to test this way: a patient cannot readily become unaware of a diagnosis, and its influence may carry over. If symptoms improve after an explanation is corrected, altered activity, concurrent treatment and natural recovery remain possible reasons alongside changed expectations. If symptoms persist, reassessment is appropriate, although persistence alone does not confirm the earlier diagnosis. The available comparisons determine how confidently a clinician can attribute the symptoms to a particular cause.

A clinician can correct misleading advice while the cause of pain remains uncertain. A claim that degeneration inevitably causes disability should be corrected because the claim is unsupported. A diagnosis that is no longer applicable and blocks a referral needs review because it affects access to care. The record can state what the patient was told, how they understood it and what has now been agreed. None of these actions require an additional diagnosis of nocebo.

### 3.6. Education About Nocebo

Spotts and Geers’ 2025 systematic review identified 21 controlled studies in 18 reports, including six experiments involving only patients. Effects varied substantially, and the authors judged the clinical evidence limited [56]. Although some studies reported promising changes in symptoms or psychological measures, these findings do not yet provide a reliable estimate of benefit for patients receiving diagnostic information.

Outcome timing and definition matter. In the headache experiment, nocebo information reduced symptoms attributed to an inert tablet after two minutes, but not after four days [57]. This measure cannot distinguish changed experience from changed attribution or reporting. In the chemotherapy trial, the seven-symptom score favoured education at the assessment scheduled for 12 weeks (difference: 4.04/70 points; 95% CI, 0.72–7.36), but not at ten days [58]. Variable completion times, 30% attrition, retrospective registration and a different pattern in clinician-rated toxicity limit interpretation.

These treatment studies leave the effectiveness of nocebo education during diagnostic disclosure uncertain. Expert recommendations support an honest discussion of expectation effects [59], without implying that patients caused their symptoms or should stop reporting them. New or changing findings still require reassessment.

Potential trade-offs extend beyond symptom outcomes. In two preregistered online experiments with adult community samples (n = 220 and 252), nocebo education lowered global side-effect expectations while increasing stated intentions to avoid further side-effect information [60]. These were immediate responses to hypothetical treatment scenarios, not observed clinical avoidance or improved informed decisions. The findings identify a possible information-related trade-off requiring assessment, rather than demonstrating clinical harm or symptom benefit. Figure 1 places expectation-related effects alongside understanding and the coordination of care.

## 4. Explaining Findings and Taking Responsibility for Care

Here, appropriate care is a context-sensitive judgement, not a synonym for more intervention, adherence or guideline compliance. Guideline-concordant care follows recommendations for a relevant population; clinically indicated care applies evidence to the individual’s findings, comorbidities and benefit–harm balance. Patient-preference-concordant care reflects an informed, voluntary choice among reasonable options. Under diagnostic uncertainty, a justified plan may involve proportionate investigation or observation, a provisional explanation, safety-netting and reassessment. These dimensions can diverge, so the reasons for the plan and any departure from guidance should be explicit. Completion means that an indicated, agreed action occurred or a justified decision not to act was recorded; inability to access a service is not an informed refusal. This operational definition guides the synthesis rather than supplying a validated appropriateness score.

### 4.1. When a Diagnosis Is Reconsidered

When a diagnosis is reconsidered, the clinician may be correcting an unwarranted label, recognising remission or interpreting a finding differently. The explanation should make clear which of these has occurred and what was known when the original judgement was made. Work on dediagnosing and the ethics of withdrawing diagnoses recognises that patients may still need the explanation and support that a label provided [61,62]. Changing the record alone may leave earlier advice in place or fail to restore access to a service.

Correction is not necessarily reassuring. Conceptual and ethical work identifies possible losses of an explanatory identity, recognition or support when a diagnosis is withdrawn [61,62]. Anxiety about an unresolved problem or mistrust of changing advice is also a potential concern, not incidence estimates established by these analyses. A pragmatic response is to explain the evidence for revision, acknowledge the implications of earlier advice and clarify which care and support remain available. Revising the diagnosis must not imply that the symptoms are disbelieved; continuing or new symptoms still require clinical assessment.

**Figure 2:**
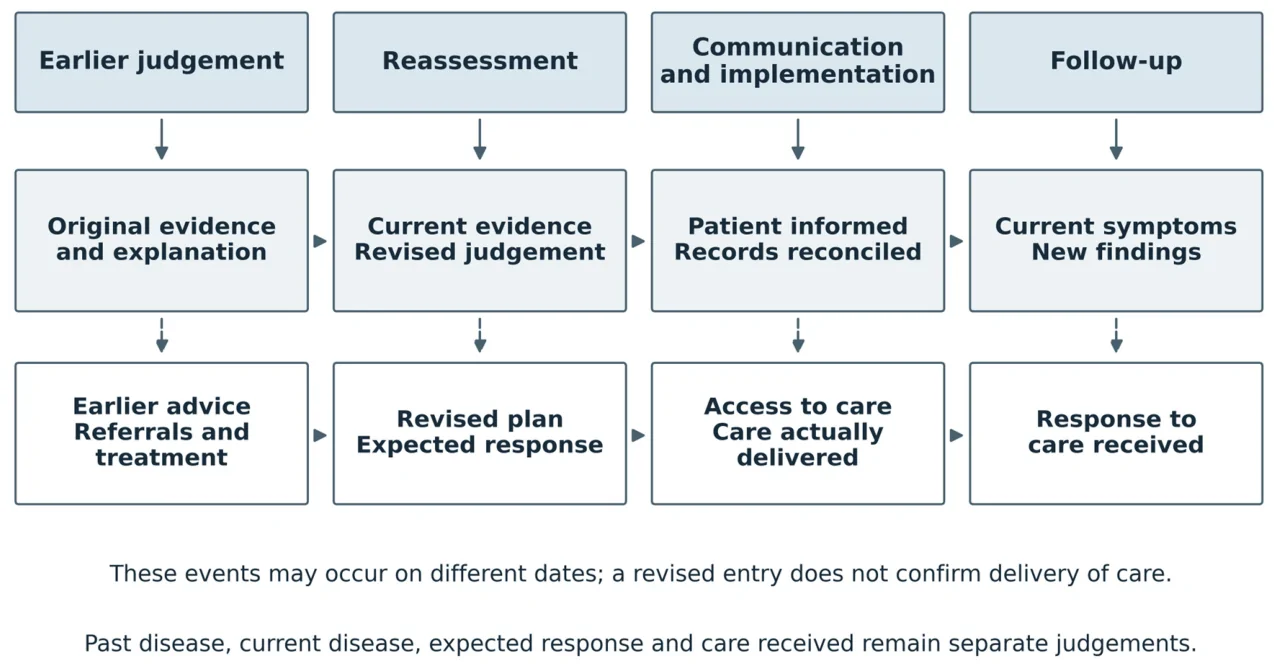
What to check after diagnostic reassessment. The upper sequence follows clinical judgements and communication; the lower sequence follows advice and care received. Dashed links mark relationships that need checking, and the events may occur on different dates. Updating a record does not confirm that advice or treatment changed. Current findings also cannot, by themselves, determine whether an earlier diagnosis was justified. This is an author-derived conceptual framework, not a validated clinical algorithm.

Reassessment begins with three questions: what supported the earlier diagnosis, what do current findings show, and what care has the patient actually received? If the original test is missing, the documentation or diagnostic process needs review. A negative test today addresses current disease within the test’s limits; it does not establish that disease was absent years ago. Similarly, a treatment the patient could not access has not had an opportunity to work. Depending on the answer, the clinician may need to retrieve earlier evidence, investigate the present symptoms or address a barrier to treatment. Figure 2 shows why the dates of revised advice and care received need to be recorded alongside the change in diagnostic judgement.

Structured reassessment in an asthma study excluded current disease in 203 of 613 evaluable adults with physician-diagnosed asthma (33.1%; 95% CI, 29.4–36.8%), after gradual medication withdrawal where relevant. At 12 months, 181 (29.5%; 95% CI, 25.9–33.1%) still had no clinical or laboratory evidence of asthma. Among participants with available community-testing data, earlier assessment of airflow limitation was documented in 64/146 (43.8%) whose current asthma was excluded and 179/322 (55.6%) whose asthma was confirmed. Twelve participants (2.0%) had serious cardiorespiratory conditions that had been misdiagnosed as asthma [63]. Reassessment thus identified missed alternatives as well as diagnoses that were no longer supported. For some patients, remission could explain why current testing did not confirm the earlier diagnosis.

### 4.2. From Revised Records to Revised Care

After a negative penicillin challenge, the allergy record may remain unchanged and the patient may still believe they are allergic. Lo and colleagues reviewed 200 drug-challenge notes from 197 patients and found 14 disagreements between challenge results and the allergy list: a total of 13 negative challenges with a retained entry and one positive challenge without an entry [64]. Later reactions could justify retaining some warnings. In the six-month PALACE follow-up, 23 of 351 respondents (6.6%) still considered themselves allergic, and 34 (9.7%) reported avoiding penicillin [65]. The investigators did not examine reasons for avoidance or audit the records, and some participants reported new adverse reactions, including immune-mediated events.

Before removing a retained allergy entry, the clinician needs to check why it remains. A patient who avoids the drug may be responding to the old label, a later reaction or current advice. Reviews describe the difficulty of maintaining de-labelling across services and the importance of informing patients and subsequent prescribers [66,67]. In a 2026 prospective cohort, 117 of 141 patients were de-labelled. Among the 88 de-labelled patients with complete follow-up, 15 (17%) were incorrectly re-labelled within twelve months despite an integrated electronic record system [68]. The study does not establish which communication change would prevent this. It does show why both the test result and the subsequent history need to be available when another clinician makes an antibiotic decision.

Randomised studies distinguish label removal from prescribing and clinical benefit. ALABAMA increased first-line penicillin prescribing after an assessment pathway combining behavioural support and record updating, but treatment response failure did not differ significantly [34] (Table 1). Prescribing became the primary endpoint after a pandemic-related amendment. Exclusion of histories consistent with anaphylaxis or serious cutaneous reactions limits application; the trial does not justify indiscriminate challenge or label removal.

PROPEL increased label removal before discharge, without significant adjusted penicillin-use differences during admission or at later assessments [35] (Table 1). These trials support different process outcomes and evaluate care packages, not communication alone.

A revised hospital record may still leave the primary-care record unchanged. In a prospective single-centre implementation study, requests to amend general-practice records were documented for 94 of 138 de-labelled patients whose primary-care records carried the allergy; records were updated in 42/94 cases (44.7%) [69]. Antibiotic exposure by WHO AWaRe category did not differ significantly between de-labelled and non-de-labelled groups. This was a short, non-randomised inpatient comparison, so the finding does not establish an absence of later prescribing benefit. Sending a result and making it available for the next prescribing decision remain separate steps.

A revised genetic classification also needs to reach the people making care decisions. The ACMG/AMP interpretation standard states that a variant of uncertain significance should not guide clinical decision-making [70]. A change in classification calls for review of the evidence and a decision about contacting the patient and revising management. Professional guidance describes responsibilities shared among laboratories, clinicians and patients [71]. The date of the laboratory update should therefore be recorded separately from patient notification and management review, so that updating a classification is not mistaken for completing follow-up.

Consider a hypothetical patient with persistent low back pain who reduced activity after being told that mild degenerative MRI findings required protection. Reassessment finds no adequate basis for that restriction, and an activity programme is agreed. At follow-up, pain and walking remain unchanged, but the referral still restricts activity and rehabilitation has not begun. The clinician needs to reconcile the advice, address access and reassess the symptoms. This differs from receiving the programme without benefit, which would prompt review of prognosis and treatment. Neither history alone establishes a nocebo mechanism. New progressive weakness or bladder disturbance would require urgent reassessment in either case.

Two further hypothetical examples illustrate how an obsolete instruction can outlast reassessment. An unreconciled asthma entry may prompt repeat medication despite an agreed, supervised change in management; the response is medication review, not unmonitored withdrawal. A copied ‘no further action’ message may prevent booking surveillance that was subsequently agreed for a pulmonary nodule. Together with the rehabilitation example, these distinguish inappropriate continuation, missed monitoring and delayed referral from failure of a treatment that was actually received. They are illustrations, not additional patient observations.

### 4.3. What a Negative Test Excludes

An angiogram showing no obstructive disease does not exclude every coronary cause of chest pain. Microvascular dysfunction and vasospasm remain possible, and consensus guidance on ischaemia with non-obstructive coronary arteries addresses their assessment [72]. Telling a patient that the heart is completely normal may imply that more has been excluded than the test allows and discourage further assessment of continuing angina.

CorMicA and ILIAS ANOCA found improvements in angina-related health status after disclosure of coronary function results with tailored management [30,31] (Table 1). CorMicA did not show a significant difference in major adverse cardiac events. The symptom benefit therefore should not be presented as established prevention of clinical events.

CorCTCA improved diagnostic classification, its primary outcome, and global treatment satisfaction, but did not demonstrate an angina benefit [32]. The adjusted symptom estimates and confidence intervals are in Table 1. Questionnaire follow-up was incomplete: a total of 140 of 231 participants at six months and 130 at one year. Neither equivalence nor a between-trial difference follows from the contrast in statistical significance.

Because both groups in CorMicA and ILIAS ANOCA underwent coronary function testing, their benefits concern disclosure with results-guided management, not testing alone or explanation separated from treatment. Differences from CorCTCA require attention to the populations, follow-up and care provided; statistical significance in one trial and not another does not itself establish a between-trial difference. Patients reassured about obstructive disease still need their continuing symptoms to be assessed on their merits.

### 4.4. Truthful Disclosure and Uncertainty

The ethical argument is distinct from the empirical question of which explanation improves outcomes. Patients need material information about their choices, including uncertainty and the consequences of waiting. The responsibility to disclose it does not depend on symptom improvement or immediate relief of anxiety. Ethical analyses of nocebo and consent resist using possible nocebo effects to justify concealment [59,73]. These are normative grounds for disclosure, not findings that disclosure necessarily reduces distress.

Empirical research instead informs how risks and options can be communicated. Reviews support clear numerical formats and attention to health literacy [74,75,76]. Where reliable absolute estimates exist, consistent denominators and time periods and complementary event/non-event formats can aid interpretation. This evidence concerns communication methods; the goal of informed, voluntary choice is an ethical commitment, and the patient’s choice may differ from the clinician’s preference.

Health literacy concerns understanding and using health information, whereas numeracy includes interpreting probabilities, denominators and time horizons. The same term, such as ‘low risk’, need not convey the same prognosis across patients. The review of risk communication for limited health literacy cautions against verbal descriptions alone and notes that graph literacy matters when visual displays are used [75]. Materials should therefore be paired with a check of the patient’s interpretation, rather than assuming that simpler wording or an illustration guarantees comprehension [76].

Evidence on positive framing comes mainly from studies of treatment side effects, and the results vary [77]. Diagnostic findings cannot always be presented reassuringly. Describing a finding as common says something about its prevalence, but does not settle its significance for this patient. Similarly, a ‘low-risk’ lesion needs an explanation of the outcome being estimated, the population on which the estimate is based and the period it covers. A clinician can recommend observation while acknowledging what remains uncertain.

Uncertainty should be made specific: whether disease is present, whether a finding explains the symptoms, what the prognosis is, or which action is preferable. A pragmatic explanation distinguishes what the test excludes from what remains possible, describes the basis for a provisional plan and identifies changes that would prompt reassessment. When a reliable probability is unavailable, a precise number should not be invented. Explaining uncertainty may alter expectations and perceived severity; its effects on trust cannot be assumed to be uniformly reassuring (Section 4.6).

A change in terminology should have a reason that can be explained to the patient. NIFTP followed a recognised pathological reclassification with defined criteria [16,17]. Replacing an unchanged cancer diagnosis with a less alarming word solely to influence treatment preferences raises a different ethical concern. The thyroid and DCIS literature studies support discussing differences in disease behaviour and the available management options, but does not establish which naming policies improve informed decisions [10,18,78]. The conversation should acknowledge that uncertainty.

### 4.5. Taking the Patient’s Account Seriously

A nocebo explanation can dismiss a patient’s symptoms if it is offered with more confidence than the evidence allows. Research on epistemic injustice describes how prejudice can lead patients’ accounts to be discounted and make their experiences harder to express in clinical terms [79]. Diagnostic overshadowing presents a related danger: an existing mental-health diagnosis can draw attention away from physical illness [80]. Suspecting that expectations contribute to symptoms is therefore a reason to examine other explanations carefully, not to stop considering them.

Taking a patient’s symptoms seriously does not require certainty about their cause. Pain can be real and disabling while its mechanism remains unclear, and a clinician can question an interpretation of a scan while acknowledging the patient’s limitations. Asking what the original diagnosis led the patient to expect may uncover a specific fear of paralysis, infertility or cancer progression. That fear gives the clinician something precise to discuss against the available evidence. A general instruction not to worry leaves the concern unanswered.

Patients who can read their records have an opportunity to identify errors and question interpretations. Ethical work on open notes argues that restricting access can reinforce unequal control over clinical knowledge [81], while portal surveys find that many respondents want immediate access even to potentially concerning results [41]. Access also depends on whether patients can use the information and obtain help. Services need to provide explanations and follow-up for people facing literacy, language or digital barriers; sending a report alone does not show that its implications have been understood.

### 4.6. Agreeing a Plan and Reviewing Progress

The consultation needs to connect the result with a decision: how well does the finding explain the presenting problem, what is still uncertain and what should happen next? The patient can then describe their understanding and say whether the proposed plan is feasible. A small pulmonary nodule, a new cancer diagnosis and an allergy label removed after testing will each require a different emphasis. Table 2 separates the evidence supporting each clinical premise from possible applications proposed by the author; these are not graded recommendations or a tested care bundle.

Questions about the next step can reveal misunderstandings that a general expression of satisfaction will miss. A patient may have heard ‘low risk’ as ‘no risk’, or ‘no urgent investigation’ as ‘no further care’. Asking why a finding is being monitored, or how the patient would seek help if symptoms changed, allows the clinician to clarify the point and check that the contact route is usable. Health-literacy and safety-netting research supports these aims [76,82], although the combination of questions proposed here has not been tested as an intervention.

Decision aids offer one established way to help patients consider evidence alongside their values. The 2024 Cochrane review, with searches through March 2022, included 209 studies and 107,698 participants and found better knowledge and more accurate risk perceptions. In 21 studies involving 9377 participants, care choices were more likely to agree with informed values (risk ratio, 1.75; 95% CI, 1.44–2.13; moderate certainty). Decision regret did not differ significantly [83]. These findings support choosing an aid evaluated for the particular decision. They do not establish that a new communication checklist will have the same benefits or improve symptoms.

Communication is an interaction, not only a report format. NICE shared decision-making guidance recommends agreeing priorities, allowing questions, giving information in manageable portions, checking understanding and providing an accessible summary and contact route [84]. For diagnostic disclosure, this supports an attentive, non-dismissive discussion of what concerns the patient. Written information and suitable visual aids can support, but not replace, that discussion. If the available time is insufficient, further discussion should be arranged rather than treating information delivery as demonstrated understanding. These are practice implications of guidance, not a tested communication bundle.

Empirical findings also caution against promising that disclosure will preserve trust. In two US online ear-infection vignette experiments, communicating diagnostic uncertainty reduced expectations of antibiotics [85]. Trust ratings were lower in the first experiment; the uncertainty comparison was not statistically significant in the second, where treatment recommendations and resistance information were also provided. That contrast neither establishes equivalence nor isolates a protective effect of adding a plan. Immediate hypothetical responses do not demonstrate actual prescribing, adherence or lasting trust. The findings are a reason to assess communication trade-offs, not to conceal uncertainty or prescribe solely to maintain confidence.

The agreed plan should describe the expected course, changes that warrant reassessment, how to obtain help and who will review unresolved findings [82]. After a diagnostic revision, it should also name the clinician or team responsible for updating records, prescriptions and referrals, with a clinically appropriate review date and an agreed handover. As a practical proposal, completion should mean that the next relevant record and decision have been checked, not merely that a message was sent. A documented, justified decision not to intervene can also complete follow-up. Patients should have a route back to care when symptoms continue or arrangements fail, rather than being left to reconcile conflicting professional advice.

The suggested applications require adaptation to the individual presentation, relevant guidance and informed preferences; the complete combinations have not been tested. Professional guidance is distinguished from trial evidence. Updating records or improving access does not establish the cause of symptoms.

### 4.7. Communication Needs and Equitable Follow-Up

Communication needs and access require separate assessment. Language, literacy, cognitive impairment and sensory needs should prompt assessment of the support required for participation, not automatic exclusion from decisions. Possible supports include an interpreter, accessible materials, repeated discussion and involvement of a trusted person in accordance with the patient’s wishes [84]. Economic circumstances, transport, time away from work and digital access should also be checked when arranging follow-up. A patient may understand a plan yet be unable to obtain the agreed care. These are practical checks, not evidence that a single adaptation works equally for every group.

Two observational studies provide context for this distinction. Cao and Pan analysed inpatient expenditure across 149 hospital service areas in Sichuan using discharge data from 2017 to 2019; expenditure was associated with demand- and supply-side factors [86]. Wang and colleagues examined healthcare-seeking and transport in 801 adults with hypertension and/or diabetes surveyed in two Enshi counties in May 2019, identifying access differences between poverty groups [87]. Their area-level and self-reported findings, respectively, do not establish the appropriateness of individual decisions or the effect of diagnostic communication. These setting-specific associations support examining access and resource use separately from understanding, without assuming that lower expenditure or fewer visits represent better care.

## 5. Research Priorities and Limitations

Future trials should study patients receiving their own results and state exactly what changed: report wording, clinician explanation, treatment or follow-up arrangements. The primary outcome should match the target problem. Accurate understanding and informed, value-consistent choices need direct measurement; appropriate care requires checking of what was actually received. Pain, function and distress should be measured separately at suitable intervals. Where scales permit, investigators should prespecify a meaningful difference and report its confidence interval, rather than equating a non-significant result with no benefit. Fewer tests or treatments count as a benefit only when necessary care is preserved, making delayed diagnosis and other safety outcomes essential.

Outcome timing needs equal attention. Early anxiety after labelling and responses to nocebo education have not followed a uniform longer-term pattern [14,57,58]. Evaluation should distinguish immediate comprehension from retained understanding, and assess adherence, healthcare use, symptoms and trust over appropriate follow-up. Dates of explanation, record correction and actual treatment initiation should be recorded separately. The relevant exposure is the information and care received, not merely when a message was sent. These are proposed measurement priorities, not a claim that communication produces a fixed sequence of outcomes.

Testing a proposed mechanism requires more than showing that communication and symptoms changed together. Expectations, perceived threat and understanding need to be measured before the outcomes they are thought to explain. Randomisation can support an estimate of a communication-and-treatment package’s total effect, but does not isolate the explanation. In observational or before–after comparisons, treatment selection and disease severity may confound associations, while natural recovery provides another explanation for improvement. Separating components requires a design that holds management comparable or varies the components independently. Even a fall in pain alongside a change in expectations does not establish mediation. Interviews can add detail about what participants understood and whether the information influenced a real decision.

Studies of diagnostic revision could begin with a chronology: when was the patient reassessed, when were they told, when were relevant records corrected and what clinical action followed? At each stage, investigators could compare the advice with the evidence available and establish whether the patient could obtain the agreed care. An appropriate decision not to intervene should count as completed follow-up. New reactions, new disease and patient preferences that justify a change in plan also need to be recorded. This approach would assess care after a correction without presuming that continuing symptoms arose through nocebo. It should capture any loss of support after a diagnosis is removed, as well as the avoidance of unnecessary treatment.

This purposive review does not provide an exhaustive or pooled estimate of clinical benefit. Its interpretive selection of the English-language literature cannot establish representativeness. The original reliance on PubMed-indexed records may under-represent psychology, communication science and health-services research; supplementary web discovery does not replace dedicated searches of those disciplines’ databases. Selection and interpretation were not independently duplicated, and formal risk-of-bias assessment was not undertaken. Hypothetical scenarios, healthy volunteers, short follow-up and interventions combining communication with other care limit clinical generalisation. The analytic framework and suggested clinical applications are author-derived and require evaluation in their intended settings.

## 6. Conclusions

Diagnostic communication can change what people understand and choose, but the evidence depends on who receives the information and what else changes in their care. Hypothetical terminology experiments, trials of management guided by results, and interventions that update records answer different questions. Experimental nocebo findings establish possible symptom mechanisms; they neither diagnose an individual patient’s symptoms nor explain failures of follow-up. The reviewed evidence does not support a single effect estimate or a universal symptom benefit from better explanation.

The practical conclusion is to identify the problem that can be demonstrated. A patient may have misunderstood a prognosis, a referral may preserve an unsupported restriction, or a revised result may not have reached the next clinician. Each calls for a specific correction, followed by assessment of whether understanding or care changed. Continuing symptoms still require clinical review; their cause is not settled by either successful communication or its failure. Correcting inaccurate diagnostic information and ensuring truthful disclosure and appropriate follow-up remain clinical responsibilities even when symptom outcomes are uncertain.

## Figures and Tables

**Table 2:** Evidence and suggested clinical applications. The ‘Evidence and Limits’ column identifies the type and limits of the cited evidence; ‘Suggested Application (Author’s Synthesis)’ contains the author’s pragmatic synthesis, not a set of validated recommendations. Evidence for a problem does not establish the effectiveness of every proposed response.

Clinical Situation	Evidence and Limits	Suggested Application (Author’s Synthesis)
Degenerative spinal imaging [3,19]	Systematic reviews of observational imaging and communication studies: abnormalities alone do not establish an individual pain mechanism; communication-effect evidence is limited.	A possible response is to relate the finding to history and examination, reconsider unsupported restrictions, and check that revised advice reaches the next clinician.
Low-risk thyroid pathology [10,16,17]	Hypothetical randomised terminology study; postoperative cohort and pathology review: naming effects differ from formal reclassification under diagnostic criteria.	A possible response is to verify the classification, explain prognosis and options, and distinguish a changed pathological diagnosis from a wording change.
Pulmonary nodule surveillance [36,45]	Patient survey and observational implementation study: distress, notification and completeness of follow-up plans are distinct outcomes; these studies do not establish mortality benefit.	A possible response is to explain surveillance, agree a modality and interval with a responsible team, and check completion or document a justified alternative.
Negative penicillin challenge [34,35,64,65,69]	Randomised care-package trials and observational follow-up: label removal, patient belief and prescribing can diverge. Later reactions may justify reassessment.	A possible response is to reconcile the test result, later reactions and records across services, and clarify continuing avoidance with the patient; challenge eligibility requires clinical assessment.
Asthma not confirmed on reassessment [63]	Prospective reassessment cohort: current non-confirmation cannot distinguish every original misdiagnosis from remission; serious alternative disease was also identified.	A possible response is to retrieve the initial evidence, consider remission and alternatives, and agree monitoring appropriate to the current findings.
Genetic variant reclassification [70,71]	Professional standards and recontact guidance: a variant of uncertain significance should not guide clinical decisions; reclassification raises notification and management responsibilities.	A possible response is to allocate patient contact and management review, recording those actions separately from the laboratory update. This operational sequence is the author’s synthesis.
Angina without obstructive coronary disease [30,31,72]	Randomised results-guided management trials and expert consensus: a negative anatomical test does not exclude all coronary causes; trials do not isolate explanation.	A possible response is to explain what the test excludes, reassess continuing symptoms and consider coronary-function evaluation in the relevant clinical setting.
Symptoms persist after a revised explanation [53,82]	Clinical review and realist synthesis of safety-netting: ongoing assessment and follow-up are relevant; no trial validates the combined checks proposed here.	A possible response is to review new findings, access and care actually received, then agree responsibility and timing for reassessment without presuming a nocebo cause.

## Data Availability

No new patient-level data were generated or analysed. The published sources discussed in this review are listed in the References section.

## Supplementary Materials

The following supporting information can be downloaded at https://www.mdpi.com/article/10.3390/diagnostics16193211/s1, Supplementary File S1: Search and evidence-selection approach (search documentation updated on 30 September 2026).
