# Supplementary material for: Diagnostic Communication and the Care That Follows: A Critical Narrative Review
Source: Diagnostics (Basel). 2026 Oct 3;16(19):3211. doi: 10.3390/diagnostics16193211 (PMC13663910; doi:10.3390/diagnostics16193211)
Supplement: Supplementary file 1 [file diagnostics-16-03211-s001.zip › diagnostics-4616131-supplementary.pdf]

Supplementary File S1

Search and Evidence-Selection Approach

Diagnostic Communication and the Care That Follows: A Critical Narrative Review
Manuscript ID: diagnostics-4616131
Supplementary searches: 30 September 2026

S1. Search history and scope

The initial literature search comprised thematic searches of PubMed-indexed records through Europe PMC from August 2026 to 23 September 2026, supplemented by targeted searches and reference-list checking. The recent English-language literature was selected purposively alongside foundational work. A complete executable log of the initial queries is not available. The strings below document the supplementary searches of 30 September 2026, not the historical August–September queries.

A supplementary thematic search was undertaken on 30 September 2026 through a general web-search interface, following links to publicly indexed PubMed/PMC records, publisher pages and institutional sources. It examined the clinical contrasts, counter-examples and qualifications relevant to the synthesis. This was not an exhaustive, prospectively registered systematic search. No complete retrieval or duplicate-screening totals are reported, and no PRISMA flow is provided.

Direct queries to PubMed and Europe PMC could not be completed during the supplementary search. Retrieval of publicly indexed records through web search therefore does not constitute a direct database rerun. No direct searches of PsycINFO, CINAHL, Embase, Scopus, Web of Science or a dedicated communication-science index were conducted. Biomedical-indexing and web-ranking limitations remain.

S2. Executed thematic web-search strings

The following strings were entered during the thematic web searches. Quotation marks indicate phrase searches. No uniform date, language, study-design or statistical-significance filter was applied at the query stage. English-language published material directly relevant to the synthesis was prioritised during selection. Search results are dynamic, and not every result or page was screened.

Terminology and labelling

```
"diagnostic labelling"
"diagnostic terminology"
"diagnostic label" "null"
```

Diagnostic communication and reassurance

```
"diagnostic communication"
"imaging" "reassurance"
"Effect of providing information about normal test results"
```

Incidental findings and follow-up

```
"incidental findings" "notification"
"Impact of Early Direct Patient Notification"
```

Nocebo education and information behaviour

```
"nocebo education"
```

```
"Identifying the psychological effects of nocebo education"
"Nocebo education and the pursuit of side-effect information"
```

Revision of diagnoses and records

```
"allergy delabelling"
"penicillin allergy delabelling"
"dediagnosing"
"variant reclassification"
"variant reclassification" "recontact"
```

Negative tests, uncertainty and interpretability

```
"coronary function testing"
"diagnostic uncertainty"
"health literacy" "risk communication"
```

The query containing “null” was an additional challenge search, not an eligibility criterion. Broader theme searches were not restricted to positive, negative or statistically significant results.

S3. Evidence selection and clinical contrasts

Sources were considered for a defined contribution: a diagnostic label or explanation linked to a patient outcome; communication or transfer of test findings linked to a care process; an experimental mechanism relevant to symptom experience; or professional/ethical reasoning needed to interpret diagnostic revision. Priority for clinical-effect claims was given to directly relevant comparisons in patients, not simply to newer publications or higher citation counts. Earlier foundational examples were retained when they supplied a distinct comparison.

The clinical domains are purposeful contrasts, not sampled specialties. Terminology studies contrast perception and stated choice; imaging studies contrast explanation with recovery or utilisation; incidental findings contrast access, notification and completed follow-up; nocebo studies contrast experimentally induced responses with individual clinical attribution; de-labelling and genomic revision contrast updated information with subsequent decisions; and coronary-function studies contrast test interpretation with results-guided management.

Qualitative studies and professional guidance were retained for experience, implementation and normative questions without treating them as estimates of treatment effect. Conversely, a trial of a combined clinical pathway was not taken to validate every suggested communication or record-reconciliation step. Reports concerned only with diagnostic technology, prediction, expenditure or non-clinical labelling were not incorporated merely because they shared a keyword.

The outcome framework is an interpretive organisation of the selected literature, not a prospectively specified framework governing identification or eligibility. Findings are mapped by the outcome measured, allowing more than one domain per study. Perceived seriousness is not assessed comprehension, preference is not treatment received, and a corrected entry is not proof that care changed.

S4. Source-level checks and counter-evidence

The entries below document selected source checks from the supplementary search. They are not a complete screening register or a full-text reappraisal of every cited article. An existing study may remain in the synthesis for its specified evidentiary contribution without its original retrieval being reproduced. Reference numbers refer to the manuscript.

O'Keeffe et al. (2022). Effect of diagnostic labelling on management intentions for non-specific low back pain.

DOI: 10.1002/ejp.1981 | PubMed: 35616226.

Located through the diagnostic-labelling search. Reference 21 informs the distinction between immediate beliefs and recovery outcomes; naming and explanation varied together. Table 1 reports the diagnostic-labelling comparison.

Howard et al. (2005). Are investigations anxiolytic or anxiogenic? A randomised controlled trial of neuroimaging to provide reassurance in chronic daily headache.

DOI: 10.1136/jnnp.2004.057851 | PubMed: 16227551.

The bibliographic record was considered as a temporal counter-example to uniform reassurance. It was not included as an independent study-level discussion; the synthesis uses the broader diagnostic-testing review and distinguishes testing strategies from explanation. No independent full-text appraisal was undertaken in the supplementary search.

Petrie et al. (2007). Effect of providing information about normal test results on patients' reassurance: randomised controlled trial.

DOI: 10.1136/bmj.39093.464190.55 | PubMed: 17259186.

The bibliographic record was relevant to explanations accompanying testing, but the full text was not retrieved during the supplementary search. It was not used as an independent source for an effect estimate in the synthesis.

Loftus et al. (2024). Impact of Early Direct Patient Notification on Follow-Up Completion for Nonurgent Actionable Incidental Radiologic Findings.

DOI: 10.1016/j.jacr.2023.07.026 | PubMed: 37820835.

An exact-title search located the primary record. Reference 33 informs the comparison between notification channels and completed follow-up, including the non-uniform findings. The numerical comparisons are reported in Table 1.

Görner, Spotts and Geers (2024). Identifying the psychological effects of nocebo education: results from two pre-registered experiments.

DOI: 10.1007/s10865-024-00520-3 | *Journal of Behavioral Medicine* 47:1080–1093.

The publisher full text was examined. Reference 60 informs Section 3.6 because stated information-avoidance intentions qualify a uniformly beneficial account of education. The inference is confined to hypothetical, immediate responses, not observed clinical harm.

Spotts et al. (2026). Nocebo education and the pursuit of side-effect information: results from three experiments.

DOI: 10.1080/08870446.2026.2668115 | PubMed: 42099040.

The primary bibliographic record was located through an exact-title search. The publisher full text was not accessible. No detailed result from this source is used in the manuscript; the full-text Görner study supplies the discussion of information-related trade-offs.

Lea and Hofmann (2022). Dediagnosing – a novel framework for making people less ill.

DOI: 10.1016/j.ejim.2021.07.011 | PubMed: 34417089.

Located through the dediagnosing search, Reference 61 provides conceptual and ethical context, not empirical validation of the suggested clinical sequence.

Jani et al. (2020). Sustaining and spreading penicillin allergy delabelling: A narrative review of the challenges for service delivery and patient safety.

DOI: 10.1111/bcp.14190.

Located through the de-labelling search, Reference 66 provides implementation context. The primary trial examples distinguish label removal, prescribing and patient outcomes.

Boerhout et al. (2025). Coronary function testing vs angiography alone to guide treatment of angina with non-obstructive coronary arteries: the ILIAS ANOCA trial.

DOI: 10.1093/eurheartj/ehaf580.

The primary publication record was located through an institutional source. Reference 31 is considered alongside CorMicA and CorCTCA: differing results do not isolate communication from management or establish a between-trial contrast through statistical significance alone.

Kobayashi et al. (2024). Clinical Variant Reclassification in Hereditary Disease Genetic Testing.

DOI: 10.1001/jamanetworkopen.2024.44526.

The primary publication page was identified in the variant-reclassification search. Laboratory reclassification alone was not treated as evidence of patient notification or completed care; this source was not included as evidence of either outcome.

Richter et al. (2023). Communication of benefits and harms in shared decision making with patients with limited health literacy: A systematic review of risk communication strategies.

DOI: 10.1016/j.pec.2023.107944 | PubMed: 37619376.

Located through the health-literacy search, Reference 75 informs empirical communication methods, separately from the ethical argument for disclosure.

Russell et al. (2024). How is diagnostic uncertainty communicated and managed in real world primary care settings?

DOI: 10.1186/s12875-024-02526-x.

The primary publisher page was located and checked in the diagnostic-uncertainty searches. It was considered for context but was not cited as an additional study. Sections 4.4 and 4.6 use shared decision-making guidance and an experimental comparison to distinguish practical suggestions from measured outcomes. No numerical claim relies on this source.

Existing comparisons retained as challenges to over-generalisation

The synthesis considers the non-significant first-exposure thyroid comparisons, LIRE's primary utilisation result, PREVENT's active comparator, incomplete or mixed reassurance outcomes, the notification-channel contrast, CorCTCA's symptom findings and the divergence between record removal and prescribing in PROPEL. These are not pooled as a uniform negative result. Each is interpreted against its own comparator, endpoint and follow-up. The nocebo-education example extends consideration to information-related trade-offs without implying observed clinical harm.

S5. Principles for critical appraisal

Appraisal is question-specific and qualitative. For each inference, the relevant considerations are the population and recruitment setting, patient versus hypothetical context, allocation and blinding where applicable, comparator, information recipient, intervention content and accompanying care, outcome definition and validity, primary versus secondary analysis, follow-up interval, attrition and precision. Applicability is considered separately from internal validity.

The review does not apply a numerical quality score, formal risk-of-bias instrument, pooled weighting or GRADE assessment. It does not infer equivalence from a non-significant comparison, or the effect of explanation alone from a treatment-and-communication package. Observational findings describe associations; qualitative findings clarify experience and implementation; professional guidance and ethical arguments justify responsibilities rather than measure symptom benefit. Table 2 labels these evidentiary roles separately from the author's suggested clinical applications.

S6. Search limitations

The supplementary searches were not exhaustive. General web discovery is affected by indexing, ranking and access. Failure to retrieve a study does not establish that none exists. Relevant psychology, communication and health-services publications may remain under-represented. Neither the initial selection nor the supplementary selection and interpretation were independently duplicated. This documentation does not convert the article into a systematic review or establish complete coverage.

S7. Targeted searches on uncertainty, communication needs and access

Date: 30 September 2026. These targeted checks complemented the thematic searches described above, using web search and direct publisher, institutional and professional-guidance sources. They did not reconstruct the original queries or comprise an additional systematic database search. No complete deduplicated screening totals are reported.

S7.1. Search concepts and executed queries

Search concepts were diagnostic uncertainty and trust; health literacy and numeracy; interaction and shared decision-making; potential unintended effects; and access or resource use. The following query strings were entered as written. Exact-title and DOI searches were also used to check sources. Failed or irrelevant retrievals were not taken as evidence that research was absent.

```
diagnostic uncertainty communication patient trust randomized trial diagnostic explanation
health literacy numeracy diagnostic information understanding risk communication randomized trial
"diagnostic uncertainty" "communication" "randomized"
"health literacy" "numeracy" "diagnostic" "comprehension"
"communicating diagnostic uncertainty"
"diagnostic uncertainty" "mistrust"
"diagnostic communication" "no effect"
"Communication of benefits and harms in shared decision making with patients with limited health literacy"
"On the Ethics of Withholding and Withdrawing Unwarranted Diagnoses"
"NG197" "time"
"ng197" "1.2.10"
"ng197" "cognitive"
"s41599-024-03712-z"
```

The DOIs 10.1177/00469580231224823 and 10.1057/s41599-024-03712-z were also followed directly to the primary publications. Some broader queries retrieved unrelated material; the search combining diagnostic communication with ‘no effect’ did not identify an additional study used in the manuscript. Existing null and mixed comparisons remained in the synthesis.

S7.2. Sources and their evidentiary roles

Reference 84—NICE shared decision-making guidance

National Institute for Health and Care Excellence. Shared Decision Making, NG197 (2021).

The complete guidance was consulted through the Health and Social Care Northern Ireland-hosted PDF. The NICE-hosted page and PDF were not directly accessible during the search. Official identifier: https://www.nice.org.uk/guidance/ng197. Consulted copy: https://engage.hscni.net/site/wp-content/uploads/2023/08/Nice-Gudielines-sdm.pdf.

Included in Sections 4.6–4.7 as professional guidance for interaction, accessible information and participation, it is not trial evidence that the combined diagnostic communication suggestions improve symptoms or work equally across groups.

Reference 85—Diagnostic uncertainty experiment

Sievert et al. (2024). Communicating diagnostic uncertainty reduces expectations of receiving antibiotics: Two online experiments with hypothetical patients. DOI: 10.1111/aphw.12536.

Primary full article, including methods and results, examined at https://iaap-journals.onlinelibrary.wiley.com/doi/full/10.1111/aphw.12536.

Included in Section 4.6 to examine a trade-off across outcomes, the interpretation is restricted to immediate hypothetical responses. The different trust results across experiments do not isolate an effect of adding treatment advice and do not demonstrate equivalence or lasting clinical benefit.

Reference 86—Geographic expenditure

Cao and Pan (2024). Understanding Factors Influencing Geographic Variation in Healthcare Expenditures: A Small Areas Analysis Study. DOI: 10.1177/00469580231224823.

Primary full article examined at https://journals.sagepub.com/doi/10.1177/00469580231224823.

Included in Section 4.7 as observational context for resource use, its area-level design cannot identify the appropriateness of each patient's care or an effect of diagnostic communication. No causal or normative claim is derived from expenditure alone.

Reference 87—Access and healthcare-seeking

Wang et al. (2024). Disparity in healthcare seeking behaviors between impoverished and non-impoverished populations with implications for healthcare resource optimization. DOI: 10.1057/s41599-024-03712-z.

Primary full article examined at https://link.springer.com/article/10.1057/s41599-024-03712-z.

Included in Section 4.7 as setting-specific context for follow-up feasibility, self-reported utilisation and transport findings are not treated as evidence of individual comprehension, diagnostic communication effects or causal effects of poverty.

S7.3. Existing evidence and interpretive safeguards

Sources on low-literacy risk communication, patient decision aids and the ethics of diagnostic withdrawal were rechecked through available publisher and institutional sources. They inform distinct questions: empirical communication findings, guidance and conceptual or ethical analysis are not interchangeable. Anxiety or mistrust after diagnostic withdrawal is identified as a potential concern without an assigned incidence rate. Clinical examples identified as hypothetical are not patient observations.

Counter-evidence was considered when a source qualified an expected benefit, measured a different outcome, indicated an unintended response or left causal attribution unresolved. Inclusion was not conditional on statistical significance. Randomised package effects are distinguished from communication-only effects, and observational associations from causal conclusions. These checks broaden the contextual discussion but do not remove the coverage, language, selection or appraisal limitations described in Section 5 of the manuscript.
